# Kölliker’s organ-supporting cells and cochlear auditory development

**DOI:** 10.3389/fnmol.2022.1031989

**Published:** 2022-10-11

**Authors:** Jianyong Chen, Dekun Gao, Lianhua Sun, Jun Yang

**Affiliations:** ^1^Department of Otorhinolaryngology-Head and Neck Surgery, Xinhua Hospital, Shanghai Jiaotong University School of Medicine, Shanghai, China; ^2^Institute of Ear Science, School of Medicine, Shanghai Jiao Tong University, Shanghai, China; ^3^Shanghai Key Laboratory of Otolaryngology and Translational Medicine, Shanghai, China

**Keywords:** Kölliker’s organ supporting cells, cochlear auditory development, degeneration, trans-differentiation, hair cells

## Abstract

The Kölliker’s organ is a transient cellular cluster structure in the development of the mammalian cochlea. It gradually degenerates from embryonic columnar cells to cuboidal cells in the internal sulcus at postnatal day 12 (P12)–P14, with the cochlea maturing when the degeneration of supporting cells in the Kölliker’s organ is complete, which is distinct from humans because it disappears at birth already. The supporting cells in the Kölliker’s organ play a key role during this critical period of auditory development. Spontaneous release of ATP induces an increase in intracellular Ca^2+^ levels in inner hair cells in a paracrine form *via* intercellular gap junction protein hemichannels. The Ca^2+^ further induces the release of the neurotransmitter glutamate from the synaptic vesicles of the inner hair cells, which subsequently excite afferent nerve fibers. In this way, the supporting cells in the Kölliker’s organ transmit temporal and spatial information relevant to cochlear development to the hair cells, promoting fine-tuned connections at the synapses in the auditory pathway, thus facilitating cochlear maturation and auditory acquisition. The Kölliker’s organ plays a crucial role in such a scenario. In this article, we review the morphological changes, biological functions, degeneration, possible trans-differentiation of cochlear hair cells, and potential molecular mechanisms of supporting cells in the Kölliker’s organ during the auditory development in mammals, as well as future research perspectives.

## Introduction

Mammals are not born with a sense of hearing, which gradually matures during the 12th to 14th day of life (Geal-Dor et al., 1993). During the development of hearing, the Kölliker’s organ supporting cells play a key regulatory role (Tritsch et al., 2007; Dayaratne et al., 2014), changing cell morphology and numbers during the embryonic and postnatal periods, eventually degenerating and disappearing after the external auditory canal opens, the endocochlear potential is established, and the cochlea can receive external acoustic stimuli, and then eventually diffuse into internal sulcus cells, at which point the cochlear hearing becomes mature (Lim and Anniko, 1985). Delayed degeneration or dysfunction of Kölliker’s organ-supporting cells can lead to abnormal cochlear hearing development (Lim, 1986). In addition, Kölliker’s organ-supporting cells can be induced to differentiate into new hair cells as inner ear progenitors for hair cell regeneration, which in turn can repair damaged hearing (Chai et al., 2011, 2012; Wang et al., 2015; Cheng et al., 2017; Zhang et al., 2017, 2018; You et al., 2018). Although the Kölliker’s organ was described more than a century ago, the molecular mechanisms regulating cochlear auditory development are still far beyond understanding. In this article, we review the morphological changes, biological functions, degeneration, possible trans-differentiation into hair cells, and potential molecular mechanisms of Kölliker’s organ-supporting cells during development, as well as future research perspectives to gain insight and understanding of the role of Kölliker’s organ-supporting cells in cochlear auditory development.

## Morphological Changes in Kölliker’s Organ-Supporting Cells During Auditory Development and Possible Mechanisms

The organ of Corti consists of a chimera of hair cells and supporting cells connected in a highly ordered manner, and is an important component of the inner ear auditory receptors (Lim, 1986). At the base of the inner spiral sulcus is a single layer of cuboidal epithelium, which is derived from the degenerated Kölliker’s organ-supporting cells (Lim and Anniko, 1985).

The Kölliker’s organ is the earliest visible epithelial structure, present in the late embryonic and early postnatal periods (P0–P14), which is a marker of cochlear immaturity (Dayaratne et al., 2014). The differentiated Kölliker’s organ consists mainly of closely spaced columnar cells, and due to the dense distribution, the nucleus can appear in different areas of the cell (mostly at the base of the Kölliker’s organ), thus appearing stratified in cross-section (Hinojosa, 1977). Kölliker’s organ-supporting cells have elongated cytosomes, usually separated by an extracellular space of about 200 Å, with some extracellular gaps down to 30 Å, with bipolar or unipolar protrusions at the top of the cell and microvilli visible at the end, which were shown to be about 3–4 μm long in mice and appeared within 10 days after birth (Hinojosa, 1977). Some of these cells can be mitotically active in late embryonic and early postnatal stages and have the potential to trans-differentiate into hair cells (Wu and Kelley, 2012; Dayaratne et al., 2014).

**Figure 1:**
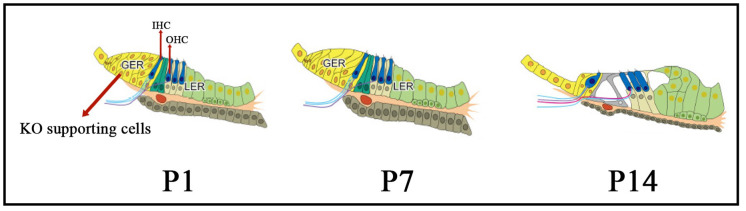
The morphology and structure of Kölliker’s organ-supporting cells from immature to mature stages. From P1 to P14, the cytoplasm moves away from the cell membrane, the cells develop folds, and the cell gaps increase in size. Also, during this critical period, the columnar cells gradually degenerate and their number decreases dramatically, and are eventually replaced by cuboidal-like cells which form a mature internal sulcus, at which point the cochlea matures in hearing. KO, Kölliker’s organ; GER, Greater epithelial ridge; LER, Lesser epithelial ridge; OHC, Outer hair cells; IHC, inner hair cells.

The morphology and structure of Kölliker’s organ-supporting cells undergo programmed changes and alterations during the embryonic period and after birth: the cytoplasm moves away from the cell membrane, the cells develop folds, and the cell gaps increase in size. After this critical period, the columnar cells gradually degenerate and their number decreases dramatically to about 12% of the original number of cells in the adult cochlea, and are eventually replaced by cuboidal-like cells that form a mature internal sulcus before degenerating and disappearing (Figure 1), at which point the cochlea matures in hearing (Tritsch et al., 2007). The origin of the cuboidal-like epithelial cells that replace the columnar supporting cells of the Kölliker’s organ during cochlear developmental maturation and remodeling of the Kölliker’s organ is unclear.

The role of ATP in initiating spontaneous morphological changes in Kölliker’s organ-supporting cells was first described by Tritsch et al. (2007). After ATP is released into the extracellular space through gap-linked hemichannels on the surface of Kölliker’s organ-supporting cells, it in turn, acts on the purine P2X and P2Y receptors on the surface of the cells themselves receptors on the cell surface, causing intracellular Ca^2+^ elevation and inward current generation (Tritsch et al., 2007; Liang et al., 2009). Using real-time imaging, the study found that these rhythmic structural changes first start in a small group of cells within the Kölliker’s organ and then radially spread to neighboring cells through gap junctions (Tritsch and Bergles, 2010). Much like inward currents, the frequency of these morphological events was significantly increased in border cells adjacent to the hair cells within the Kölliker’s organ region, suggesting that border cells around the hair cells first initiate this spontaneous activity and play a key regulatory role (Nishani Dayaratne et al., 2015).

This spontaneous morphological change in the rodent Kölliker’s organ does not occur at birth, and studies suggest that cellular morphological changes need to be initiated when the cochlea develops to a certain extent. The low level of purinergic receptor expression on the surface of Kölliker’s organ-supporting cells in the early postnatal period results in ATP-induced inward currents and calcium waves at low levels, which are insufficient to initiate this process (Tritsch and Bergles, 2010). It was found that neither strong current stimulation nor elevated extracellular K^+^ ion concentrations induced spontaneous morphological changes; whereas when the increased intracellular Ca^2+^ concentrations, cells underwent depolarization, activated Kölliker’s organ-supporting cells, and further promoted changes in cell diameter in neighboring cells (Tritsch et al., 2007; Tritsch and Bergles, 2010). These results suggest that the intracellular Ca^2+^ concentration is a key factor in triggering rhythmic morphological changes in cells that diffuse into adjacent cells within the Kölliker’s organ (Tritsch et al., 2007; Peng et al., 2012).

It is now believed that one of the key factors initiating this morphological change is the Ca^2+^-activated Cl^−^ channel, leading to Cl^−^ efflux, which decreases the cell membrane threshold potential level and further causes an efflux of intracellular fluid due to the osmotic gradient, resulting in sawtooth morphological changes in Kölliker’s organ-supporting cells (Tritsch et al., 2010). Furthermore, this outflow of intracellular fluid may be influenced by the distribution of water channel proteins (especially AQP4) within the Kölliker’s organ-supporting cells (Nishani Dayaratne et al., 2015), and additionally, the activation of intracellular contractile proteins may also be responsible for the altered morphology of the supporting cells (Tritsch et al., 2010; Liu Y. et al., 2019).

## Biological Functions of Kölliker’s Organ-Supporting Cells in The Regulation of Cochlear Hearing Development

### Intracellular spontaneous electrical activity in Kölliker’s organ-supporting cells promotes auditory maturation

Studies have shown that afferent nerve fibers are not in a silent state before the onset of hearing and that the hair cells continuously release low levels of glutamate, triggering spontaneous electrical activity that plays an important role in promoting the survival and maturation of auditory neurons, the development of synapses, and the refinement of audio localization (Lelli et al., 2009; Blankenship and Feller, 2010; Wang and Bergles, 2015). Rodents are not auditory at birth and do not respond to external sounds until 2 weeks after birth, and Kölliker’s organ-supporting cells play a key role in this auditory maturation process (Forsythe, 2007; He and Yang, 2011).

The inner and outer hair cells generate spontaneous calcium action potentials in the first week of life. Spontaneous calcium action potentials begin to guide cochlear development before the appearance of hearing (Beurg et al., 2008; Marcotti, 2012; Sendin et al., 2014). While this spontaneous calcium action potential of immature inner and outer hair cells is certainly important in cochlear development, the developmental maturation process of hearing also relies on the cellular communication network formed by Kölliker’s organ-supporting cells through gap junctions. In the second week of life, Kölliker’s organ-supporting cells begin to release adenosine triphosphate (ATP) spontaneously and release ATP to the endolymphatic surface *via* gap junction hemichannels composed of Cx26 and Cx30 between Kölliker’s organ-supporting cells and act as paracrine receptors on the surface of neighboring hair cells to produce phospholipase C (PLC)-dependent diacylglycerol and inositol triphosphate (IP3; Rodriguez et al., 2012; Giorgi et al., 2018). The intracellular diffusion of IP3 and subsequent binding to IP3 receptors in hair cells triggers the release of Ca^2+^ from the endoplasmic reticulum (ER) calcium pool and an increase in cytoplasmic Ca^2+^ concentration, which further induces the release of the neurotransmitter glutamate from synaptic vesicles in inner hair cells, excites hair cells and their afferent nerve fibers, activates type I spiral neurons (SGNs) to generate action potentials and in this way transmits cochlear development to the upstream auditory pathway. This transmits temporal and spatial information about cochlear development to the upstream auditory pathway and refines synaptic connections in the auditory pathway, thus promoting cochlear maturation and auditory acquisition (Tritsch and Bergles, 2010; Rodriguez et al., 2012; Johnson et al., 2017). It has been suggested that ATP released by Kölliker’s organ-supporting cells primarily coordinates spontaneous electrical activity with its neighboring inner hair cells, with little association with the more distant inner hair cells, and it is hypothesized that this mechanism may be critical for the establishment of audio localization in the cochlear nucleus before the emergence of hearing (Tritsch et al., 2007).

### Ca^2+^ release from Kölliker’s organ-supporting cells promotes cochlear development

Ca^2+^ is the primary messenger for intercellular messaging in the cochlea and is involved in the transduction of a large number of signaling pathways (Tritsch et al., 2010). ATP in the cochlea triggers changes in intracellular Ca^2+^ concentration in Kölliker’s organ-supporting cells and intercellular calcium wave transmission, which in turn transmits important biological information to the cochlear sensory epithelium. Organ-on-chip model studies have shown that developing cochlear Kölliker’s organ-supporting cells release ATP to drive spontaneous Ca^2+^ signals *via* gap junction protein hemichannels (Mazzarda et al., 2020). Before the appearance of hearing, the spontaneous release of ATP from the Kölliker’s organ further mediates the release of Ca^2+^ within Kölliker’s organ-supporting cells after release into the extracellular compartment *via* hemichannels, initially inducing the release of Ca^2+^ from only a small group of Kölliker’s organ-supporting cells, which subsequently propagate rapidly through the intercellular gap junction channels, further initiating the release of Ca^2+^ from the calcium pool in neighboring Kölliker’s organ-supporting cells. The release of large amounts of Ca^2+^ promotes the synchronization of neighboring cells, which further causes the release of ATP (Zhao et al., 2005). In this way, the P2 purinoceptor/phospholipase C/IP3/Ca^2+^ signaling cascade is maintained in an orderly and positive feedback manner, causing sustained spontaneous electrical activity, and promoting cochlear development.

Kölliker’s organ-supporting cells form a functional syncytium through extensive intercellular gap junctions (Goodenough and Paul, 2009), through which intercellular Ca^2+^ propagation and ATP release occur, providing a Kölliker’s organ cell-to-cell signaling metabolic basis, i.e., facilitating intercellular signaling when the gap junctions are open and closing them under some specific physiological conditions (Goodenough and Paul, 2009; Mammano, 2013). GJB2 mutations are mainly characterized by degeneration of outer hair cells and hypoplasia of the vascular stripe and are the main cause of deafness in 50% of pre-speech deafness (Hochman et al., 2010). In addition, GJB6 mutation in rodents was found to reduce Ca^2+^ in Kölliker’s organ-supporting cells, leading to an increased hearing threshold (Rodriguez et al., 2012; Chen et al., 2021a); while Cx26 cKD mice also showed reduced ATP release, downregulation of ATP-dependent calcium signaling, the disappearance of calcium waves and increased hearing threshold in Kölliker’s organ-supporting cells (Sun et al., 2022).

### Kölliker’s organ-supporting cells promote the formation of tectorial membrane in the organ of corti

Studies have shown that glycoproteins secreted by Kölliker’s organ-supporting cells, such as otocollagen and capsid protein, are involved in the formation of the tectorial membrane (Hinojosa, 1977; Lim and Anniko, 1985; Zine and Romand, 1996). During early developmental stages, the tectorial membrane is attached to the Kölliker’s organ-supporting cells by a network of thin filaments, which gradually separate as the cochlea matures, and about 2 weeks after birth, the tectorial membrane is completely separated from the Kölliker’s organ and eventually extends to the out hair cells. Thyroid hormone is a key factor in regulating this process, and thyroid hormone deficiency prolongs the survival of Kölliker’s organ-supporting cells and causes tectorial membrane malformations (Uziel et al., 1981).

PR-domain-containing 16 (PRDM16) is a key transcriptional regulator in craniofacial, adipose, and neural tissue development (Fumasoni et al., 2007; Shull et al., 2020). It was found that PRDM16 is highly expressed in the nuclei of Kölliker’s organ-supporting cells in the bottom and middle cochlear apex by embryonic day 13.5 and decreases rapidly at birth. Its expression in the Kölliker’s organ continued throughout development until postnatal day 7, and PRDM16 was thus suggested as a possible specific marker for Kölliker’s organ-supporting cells (Ebeid et al., 2022). Further, by studying the model with Prdm16cGT double allele deletion mice, it was found that the cochlear Kölliker’s organ of Prdm16 gene-deficient mice at day P0 had poor development, shortened cochlear ducts, reduced proliferation of Kölliker’s organ-supporting cells, increased density of hair cells and supporting cells in the parietal ring, and loss of anchoring ability of the lid membrane to the Kölliker’s organ, indicating that Prdm16 is a regulatory gene necessary for Kölliker’s organ-supporting cells’ proliferation (Ebeid et al., 2022). Cochlear duct development requires the proliferation of these cells for convergent extension and to achieve normal hair cells and supporting cell densities within the organ of Corti (Chen et al., 2002; Driver et al., 2017). In addition, gene expression analysis revealed that α-tectorin (TECTA) expression was downregulated and β-tectorin (TECTB) appeared upregulated in the cochlea of Prdm16cGT-deficient mice. α-TECTA and TECTB are essential proteins in the tectorial membrane (Richardson et al., 2008), and mutant mice with α-tectorin exhibit abnormal tectorial membrane and hearing loss (Legan et al., 2014; Kim et al., 2019), and the study hypothesized that deletion of the Prdm16cGT gene leads to dysregulation of tectorin subunit expression, resulting in abnormal tectorial membrane morphology.

## Kölliker’s Organ-Supporting Cells Subtypes

Single-cell RNA transcriptome sequencing-based studies have revealed that Kölliker’s organ-supporting cells also have different cellular subtypes (Chen et al., 2021b). The study, based on clustering analysis of cells with similar gene expression patterns, revealed the existence of four different cell subtypes in Kölliker’s organ-supporting cells (Kolla et al., 2020; Chen et al., 2021b; Kubota et al., 2021). The Kubota et al. (2021) study of organoid development classified Kölliker’s organ-supporting cells into three different cell subtypes. Gene expression localization showed that specific overexpressed genes were displayed in different regions of the Kölliker’s organ and gradually degenerated and disappeared as the cochlea matured (Kubota et al., 2021). Chen et al. (2021b) identified four cell subsets from P1 to P14, of which the number of cells decreased gradually, while the other four cell subsets proliferated at P1 to P7 and completely disappeared at P14. These eight cell subpopulations were found to be closely linked on the t-SEN cell space map and had very similar gene expression patterns and biological properties (Kolla et al., 2020; Chen et al., 2021b; Kubota et al., 2021). Kamiya et al. (2001) also found that apoptosis and mitosis co-exist in Kölliker’s organ-supporting cells at P7 postnatally and that the cells not only degenerate but also regenerate before disappearing around P12, demonstrating that mitotic proliferation of Kölliker’s organ-supporting cells occurs not only in embryonic development but also in the development of the normal auditory system after birth.

## Molecular Mechanisms of Kölliker’s Organ-Supporting Cell Degeneration

Impaired or delayed degeneration of the Kölliker’s organ can lead to structural deformities of the organ of Corti, especially the organ of Corti lid (Uziel, 1986). However, the molecular mechanisms of Kölliker’s organ-supporting cell degeneration are not clear.

### Apoptosis induces programmed degeneration of Kölliker’s organ-supporting cell

He and Yang (2015b) found that the morphology of Kölliker’s organ-supporting cells in the neonatal P1, P3, P5, P10, P12, and P14 rat cochlea gradually changed from short columnar to tall columnar cell morphology from the basal turn to the apical turn and the number of cells also gradually decreased in addition to the change in cell morphology, presuming that apoptosis of Kölliker’s organ-supporting cells exists during the development of the neonatal rat cochlea (He and Yang, 2015b). He and Yang (2015a,b) showed that Kölliker’s organ-supporting cells exhibited a programmed apoptotic process from basal turn to the apical turn, but *in vitro* tests showed proliferation, suggesting that the initiating factor of apoptosis may come from outside the Kölliker’s organ-supporting cells rather than from intrinsic cellular factors. On this basis, Hou et al. (2019, 2020) further found that the expression levels of caspase-3, caspase-8, caspase-9, and BCL-gene mRNA and protein in the cochlear basement membrane of rats at different days of age after birth were significantly time-dependent, and the study concluded that *in vivo* Kölliker’s organ-supporting cells not only existed in apoptosis but also in proliferation. The number of proliferating cells is much less than the number of apoptotic cells, which eventually manifests in the tissue structure as the disappearance of Kölliker’s organ-supporting cells (Hou et al., 2019, 2020).

Liu et al. (2017) investigated the morphological changes of Kölliker’s organ-supporting cells and the expression of apoptosis-related factors during early postnatal development. It was found that on day P5, the Kölliker’s organ in the basal turn of the cochlea became significantly smaller, while the Kölliker’s organ in the middle and apical turn became smaller. On day P12 the cochlea showed the disappearance of the Kölliker’s organ in the basal turn and middle turn, and at this stage, the Kölliker’s organ in the apical turn was still present, but the number of cells was significantly reduced and its morphology changed from a high columnar to a short columnar shape. The results of immunohistochemistry and TUNEL staining showed the presence of necrotic cytomorphological changes in some TUNEL-positive Kölliker’s organ-supporting cells at day P7, showing chromatin condensation and vacuole-like changes (Liu et al., 2017).

It was concluded that exogenous and endogenous apoptotic pathways exist during the apoptosis of supporting cells as the rat cochlear Kölliker’s organ develops *in vivo*. After birth, the Kölliker’s organ in the cochlea degenerates in a time-dependent pattern in which cysteine proteases are involved in apoptotic cell death during postnatal development, suggesting the involvement of endogenous factors (Adrain and Martin, 2001).

Apoptosis and proliferation co-exist during the degeneration of the Kölliker’s organ, and the degenerative disappearance of the Kölliker’s organ is thought to be a disturbance of the balance between apoptosis and mitosis (Kamiya et al., 2001). It is still unclear why such cell proliferation exists in Kölliker’s organ-supporting cells at a later stage of their degenerative disappearance.

### The regulatory role of thyroid hormone (T3) in the degeneration of the Kölliker’s organ-supporting cells

The thyroid hormone is a key factor in guiding the orderly degeneration of the Kölliker’s organ during cochlear development (Mustapha et al., 2009; Peeters et al., 2015; Sundaresan et al., 2016). Hypothyroidism and mutations in the thyroid hormone receptor beta gene cause delayed degeneration of the Kölliker’s organ, while ectopic treatment with T3 (3,5-triiodo-L-thyroxine) on days P0 and P1 leads to earlier degeneration of the Kölliker’s organ and hearing loss (Rusch et al., 2001; Peeters et al., 2015; Borse et al., 2021). Peeters et al. (2015) investigated the time course of apoptosis support by the Kölliker’s organ and the effect of ectopic T3 on apoptosis. Furthermore, in methimazole-treated hypothyroid rat cochlea studies showed that Kölliker’s organ-supporting cells lacked TUNEL-stained positive apoptotic cells at day P7. It has been reported that apoptosis in Kölliker’s organ-supporting cells may be regulated by some unknown mechanism regulating the expression of the thyroid hormone receptor β gene in Kölliker’s supporting cells and thus regulating this programmed apoptosis (Ng et al., 2013).

Studies have reported that immunoreactivity for the neurotrophin receptor p75NTR, a mediator of neuronal survival or differentiation, is detected in the Kölliker’s organ of P5 rats and that this immunoreactivity changes in response to thyroxine (T4; Knipper et al., 1999). It is unclear whether p75NTR contributes to Kölliker’s organ-supporting cell apoptosis (Sato et al., 2006). T3-induced Kölliker’s organ-supporting cells’ apoptosis is a unique cell- and time-specific form of apoptosis in the late mitotic phase of tissue maturation and may involve a series of pre-apoptotic events, including cellular reorganization and cellular translocation before the cell death phase, in addition to how this process is coordinated with other unknown signals together with other unknown signals, still need to be investigated in depth (Coen et al., 2007). Although it is not clear how premature remodeling leads to deafness, these studies suggest that cochlear development is a precisely ordered regulatory process and that T3 provides an important molecular signaling role in the orderly regulation of these remodeling events.

### Autophagy regulates the programmed degeneration of Kölliker’s organ-supporting cells

Several studies have reported the relationship between autophagy and hearing loss, and activation of autophagy can reduce noisiness (Guo et al., 2021), drug resistance (He et al., 2017; Liu et al., 2021; Zhang et al., 2022), and the degree of inner ear damage and hearing loss in the elderly (He et al., 2020, 2021). It was found that autophagy is closely related to the occurrence and development of sensorineural deafness (Zhou et al., 2020) and that autophagy activators can effectively reduce the level of oxidative stress in hair cells and decrease hair cell death, thus attenuating high-intensity noise-induced damage to hair cells (Li et al., 2018). In addition, autophagy also plays an important regulatory role during inner ear development and is essential for maintaining the morphology and function of auditory hair cells (Zhou et al., 2020).

The existence of autophagy during Kölliker’s organ-supporting cell degeneration was investigated and a large number of autophagic vacuoles containing organelles was first detected by Hinojosa (Hinojosa, 1977). Autophagy markers, including LC3-II, SQSTM1/p62, and Beclin1, were detected in Kölliker’s organ-supporting cells by immunohistochemical staining. In addition, it showed that during early postnatal cochlear development, the expression of LC3-II gradually decreased and the expression of p62 increased, compared with that between P7 and P10. Compared to apoptotic markers that peak at P1, autophagic flux peaks earlier at P1, and activated autophagic flux gradually decreases with the degeneration of Kölliker’s organ-supporting cells from P1 until P14 (Hou et al., 2019).

Apoptosis and autophagy in Kölliker’s organ-supporting cells show different time-dependent and sophisticated expression patterns, with the peak of autophagic activity during postnatal maturation of cochlear hearing development located on postnatal day 1 (P1) or earlier, while the expression of apoptotic markers (Bcl-2, caspase-3, caspase-8, and caspase-9) follows a bell-shaped curve, with peak expression at P3 (Qu et al., 2007). Organelles are digested by autophagy before apoptosis, and autophagy can provide a source of energy for the removal of aggregated proteins and damaged organelles, both of which play different roles at different stages of cochlear development (Aburto et al., 2012; Liu et al., 2017). These results suggest that autophagy plays an important early role in the pre-apoptotic transformation and degeneration of the Kölliker’s organ during early postnatal development and that any disturbance in autophagy or apoptosis of Kölliker’s organ-supporting cells during development will lead to impaired cochlear development and eventually cause hearing loss (He et al., 2017). Therefore, the dynamic balance between autophagy and apoptosis regulates the normal differentiation and orderly organization of developing cochlear supporting cells, but the regulatory mechanism of the dynamic balance between autophagy and apoptosis is currently unknown (He et al., 2017; Zhou et al., 2020). Abnormal intracellular calcium signaling mediated by Gjb2 may be a key regulator of abnormal alterations in the autophagy-apoptosis signaling pathway and still requires in-depth study (Inoshita et al., 2014; Sun et al., 2022).

### Molecular signaling pathways of Kölliker’s organ-supporting cells degeneration

Studies have confirmed that some important genes and signaling pathways play an important role in cochlear hearing development, such as Sox2 (Cheng et al., 2019), Pou4f3, and Atoh1 genes (Chen et al., 2021c); FGF (Tang et al., 2016), Notch (Ni et al., 2016; Waqas et al., 2016), FoxG1 (He et al., 2020, 2021), Strip1 (Zhang et al., 2021), mTOR (Fu et al., 2018, 2022) and Wnt signaling pathway (Chai et al., 2012; Liu et al., 2016). However, the molecular mechanisms of signaling pathways associated with the degenerative disappearance of Kölliker’s organ-supporting cells early in postnatal cochlear hearing development are not clear. It has been proposed that Hedgehog signaling inhibits the sensory differentiation ability of Kölliker’s organ-supporting cells, regulates the fate of Kölliker’s organ-supporting cells, and induces cochlear developmental maturation (Driver et al., 2008).

**Figure 2:**
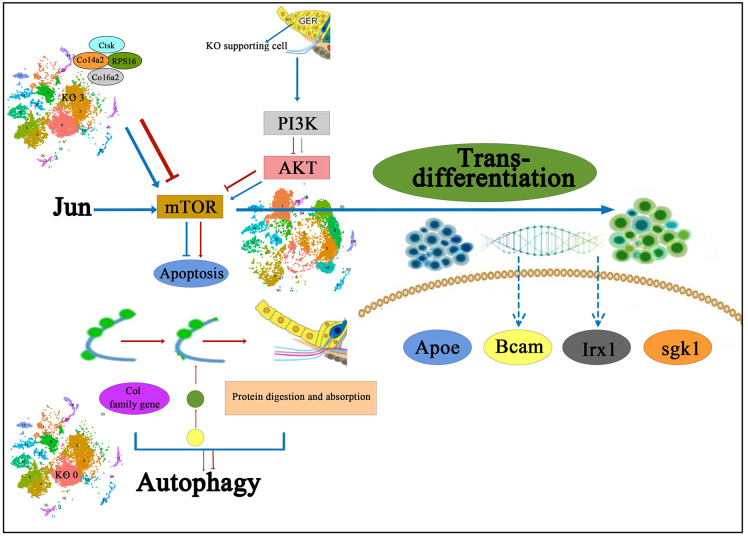
The proposed signal pathway diagram of the molecular mechanism of Kölliker’s organ-supporting cells degeneration and trans-differentiation. It is shown that Col family genes on KO 3 and the PI3K-Akt signaling pathway are presumed to regulate the dynamic balance of proliferation and apoptosis in Kölliker’s organ-supporting cells, and also the Col family genes in the KO 0 regulate the autophagy of these apoptotic proteins and cellular debris and provide the energy required to promote the apoptotic process. In addition, it has been proposed that the PI3K-Akt-mTOR signaling pathway may be one of the underlying mechanisms to induce the differentiation process of Kölliker’s organ-supporting cells to hair cells. IHC, inner hair cells; KO 0, Cluster 0 of Kölliker’s organ-supporting cells; KO 3, Cluster 3 of Kolliker’s organ-supporting cells.

Chen et al. (2021b) performed KEGG signaling pathway analysis in different subtypes of Kölliker’s organ-supporting cells by scRNA-seq and found that genes in different subtypes of Kölliker’s organ-supporting cells were significantly enriched in the Ribosome signaling pathway; however, in the Cluster 3, a large number of genes were enriched in the Ribosome signaling pathway and Protein digestion and absorption signaling pathway. The Ribosome signaling pathway is an important signaling pathway that regulates cell proliferation and development (Pelletier et al., 2018). The results of scRNA-seq showed that from P1 to P7, there was a significant proliferation of cell numbers, but cell numbers showed a decrease throughout Kölliker’s organ-supporting cells development, presumably mainly associated with the PI3K-Akt signaling pathway, a negative regulatory pathway of gene expression in the Cluster 3. The PI3K-Akt signaling pathway is an important signaling pathway that regulates cell proliferation, differentiation, apoptosis, and migration (Jia et al., 2019; Bu et al., 2022). It has also been shown to regulate cochlear hair cell regeneration (Mullen et al., 2012; Xia W. et al., 2019). A large number of Col family genes on Cluster 3 are enriched in the PI3K-Akt signaling pathway, including Col4a1, Col4a2, Col6a1, Col6a2, and Col1a1, which are presumed to regulate the dynamic balance of proliferation and apoptosis in Kölliker’s organ-supporting cells. The Col family genes Col3a1, Col5a1, Col5a2, Col6a1, and Col1a1 in the Cluster 0 regulate the autophagy of these apoptotic proteins and cellular debris and provide the energy required to promote the apoptotic process through autophagic catabolism (Komatsu et al., 2005), which in turn directs the Kölliker’s organ-supporting cells under the sequential degeneration or trans-differentiation of Kölliker’s organ-supporting cells. The role of this signaling pathway and related genes in regulating Kölliker’s organ-supporting cell degeneration and auditory development needs to be further investigated. The proposed signal pathway diagram of the molecular mechanism of degeneration and trans-differentiation of Kölliker’s organ-supporting cells has been shown in Figure 2.

## Kölliker’s Organ-Supporting Cells Have The Potential to Trans-Differentiate into Hair Cells

Adult mammalian cochlear hair cells have no regenerative capacity, but studies have shown that newborn rodent cochlear basement membrane cells have a limited but transient regenerative potential, which is mainly derived from non-sensory cells in the cochlea (Chai et al., 2018). Under normal conditions, mitotic cells in the neonatal mouse cochlea are relatively quiescent, and early damage caused by various factors will indirectly activate these non-sensory cells to exert their limited regenerative potential to restore the damaged hair cell population and thus repair hearing (Gao et al., 2019; Liu W. et al., 2019; Chai et al., 2022). Kölliker’s organ-supporting cells normally exist in the late mid-embryonic and early postnatal stages as a transient neonatal cell population, and it was found that some cell populations located in the Kölliker’s organ region retain the properties of precursor cells, which can regenerate and transform and are the precursor cell pool for hair cell regeneration (Chai et al., 2011, 2012; Bramhall et al., 2014).

### Math1 overexpression induces the trans-differentiation of Kölliker’s organ-supporting cells into hair cells

Math1 is a homolog of the Drosophila gene, the deletion of which leads to failure of inner ear hair cell differentiation (Chen et al., 2002). It was found that induction of Math1 overexpression in Kölliker’s organ-supporting cells led to the generation of a large number of ectopic hair cells, and these newborn ectopic hair cells were positive for myosin VIIa (a hair cell-specific marker) and could form keratin plates and stereocilia bundles (Zheng and Gao, 2000; Zhang et al., 2007).

Overexpression of Math1 resulted in the interdental cells of the organ of Corti and its adjacent organ of Corti, the internal sulcus, and the Hensen’s cells regions, and axonal extension from the auditory nerve bundle to some of the new hair cells regions, suggesting that the new hair cells attract the guided regeneration of auditory spiral neurons (Gubbels et al., 2008).

The Kölliker’s organ does not express the Math1 gene after birth and is normally expressed only in the embryonic sensory epithelium starting at E12.5, suggesting that the reason why Kölliker’s organ-supporting cells do not continue to differentiate into hair cells after birth but gradually transform into internal sulci, most likely because of the absence of Math1 expression (Bermingham et al., 1999). Math1 is expressed in differentiated hair cells in the early embryonic stage but is downregulated in supporting cells in the late embryonic stage and is absent in cells outside the sensory epithelium (Chen et al., 2002; Woods et al., 2004). In addition, it was found that when cochlear organ cultures overexpressing Math1 caused the production of ectopic hair cells within the large and small epithelial crest regions, while knockdown of Hes1 also resulted in the production of ectopic hair cells and these hair cells showed elevated levels of Math1 expression (Zheng and Gao, 2000). The Hes1 gene is normally expressed only in the Kölliker’s organ of P0–P3, suggesting that Hes1 may repress Math1 expression to some extent early in life, consistent with the idea that Hes5 is primarily involved in the precise regulation of ectopic hair cells (Zine et al., 2001; McGovern et al., 2018). Math1 is an essential gene for hair cell differentiation and plays a positive regulatory role, while Hes1 and Hes5 are negative regulators of hair cell differentiation (Zine et al., 2001; Su et al., 2015). Future attempts might be made to continue the differentiation of postnatal Kölliker’s organ-supporting cells to hair cells by upregulating the expression levels of Math1 and or downregulating Hes1 in Kölliker’s organ-supporting cells.

### Atoh1 overexpression induced trans-differentiation of Kölliker’s organ-supporting cells into hair cells

It was found that after induction of Atoh1 overexpression, ectopic hair cell regeneration occurred in different subtypes of Corti organelle supporting cells (Liu et al., 2012; Richardson and Atkinson, 2015; Walters et al., 2017; Yamashita et al., 2018). Kelly et al. (2012) identified a large number of isolated ectopic hair cells in the non-sensory Kölliker’s organ region by constructing a triple allele TGATOH1 mouse model. These ectopic hair cells displayed a stereocilia structure similar to that of mature hair cells, but exhibited different maturation and were scattered within the Kölliker’s organ (Kelly et al., 2012). Ectopic hair cells regeneration occurs most vigorously in the Kölliker’s organ region, but the efficiency of induced transformed hair cells remains low, compared to ectopic expression of Atoh1 alone, and it was shown that co-activation of Atoh1 with other transcription factors, such as Pou4f3, Gfi1, Gata3, and Nymc, induces more stem cells to trans-differentiate into hair cell-like cells, and the newly generated cells are more mature in both neonatal and mature mouse cochlea (Liu et al., 2012; Walters et al., 2017; Chen et al., 2021c). Xu et al. (2021) induced Atoh1 in Sox2-overexpressing positive progenitor cells in Kölliker’s organ and found that ectopic expression of Atoh1 promoted the trans-differentiation of progenitor cells to hair cells regeneration while demonstrating the importance of the Isl1/Tub/Znf532 pathway in promoting Atoh1-mediated hair cells regeneration. All these studies suggest that the supporting cells in the lateral of the Kölliker’s organ can serve as a source of progenitor cells for hair cell regeneration in the early postnatal period.

### Lgr5-positive progenitor cells in the Kölliker’s organ have the potential to generate cochlear hair cells

In the neonatal mouse cochlea, Wnt-responsive Lgr5-expressing cells are progenitors of regenerative hair cells, and upregulation of Wnt signaling stimulates the proliferation of Lgr5-positive progenitors (Chai et al., 2011, 2012). Lgr5 expression decreases gradually during cochlear development and maturation, mainly in IPs, and IPCs. In the adult cochlea, Lgr5 is only expressed in D3 s (Chai et al., 2012). Several studies have shown that Lgr5-labeled cochlear progenitor cells, following hair cell injury, can regenerate by mitosis and/or directly differentiate to generate new cochlear hair cells in the early postnatal period (Shi et al., 2012, 2013; Cox et al., 2014).

Recent studies have shown that multiple signaling pathways play an important role in hair cell regeneration by inducing proliferation and differentiation of Lg5 progenitor cells (Chen et al., 2021d). Activation of Wnt/β-linked protein signaling and inhibition of Notch signaling can induce Lgr5 progenitor cells to regenerate Myo7a-positive hair cells (Chai et al., 2012; Korrapati et al., 2013; Mizutari et al., 2013). Lgr5 progenitor cells can be regulated by many other factors and signaling pathways such as Shh, Foxg1, and Hippo (Gregorieff et al., 2015; Chen et al., 2017; Cheng et al., 2017; Zhang et al., 2020). The efficiency of regenerating hair cells from inner ear Lgr5 progenitor cells remains very limited, suggesting that hair cells regeneration involves other factors or signaling pathways (Wu et al., 2016; Lu et al., 2017; Fang et al., 2019; Xia L. et al., 2019). A previous study of hair cell regeneration and differentiation in the presence of SEC inhibitor flavopiridol showed that flavopiridol decreased the proliferative capacity of Lgr5 progenitor cells, but with no effect on the differentiation ability (Chen et al., 2021d). These results suggest that SEC plays an important role in regulating inner ear progenitor cells and thus influencing hair cells regeneration, and further *in vivo* studies are needed to elucidate the role of SEC in the inner ear, which will lay the experimental foundation for using cochlear progenitor cells to regenerate functional cochlea for the treatment of patients with sensorineural deafness.

Zhang et al. (2020) studied using Sox2^CreER/Foxg1loxp/loxp^ mice and Lgr5^EGFPCreER/Foxg1loxp/loxp^ mice to conditionally and specifically knock down Foxg1 in Sox2supporting cells and Lgr5 progenitor cells of neonatal mice, respectively and found that conditional knockdown of Foxg1 in both Sox2 supporting cells and Lgr5 progenitor cells at postnatal day 1 (P; cKD) resulted in the appearance of a large number of additional hair cells that In particular, more additional internal hair cells were generated at day P7, and these nascent hair cells with normal cilia bundles and synapses survived until at least P30. In addition, sphere formation assays showed that Foxg1 cKD in Lgr5 progenitor cells did not significantly alter their sphere formation capacity, and the findings suggest that Foxg1 cKD may directly trans-differentiate supporting cells and progenitor cells by inducing This study provides new evidence for the role of Foxg1 in regulating hair cell regeneration from supporting cells and progenitor cells in the neonatal mouse cochlea (Zhang et al., 2020).

### BMI1 and cochlear hair cell regeneration

It was shown that Bmi1 regulates redox homeostasis and reactive oxygen species (ROS) levels and is expressed in hair cells and supporting cells in addition to spiral ligament and spiral ganglion cells, thus playing an important role in the survival of auditory hair cells (Chen et al., 2015). It was found that Bmi1 knockdown significantly reduced the sphere formation ability of neonatal mouse Corti organ-supporting cells and Lgr5-positive progenitor cells, suggesting that Bmi1 is required to initiate the proliferation of neonatal mouse supporting cells and progenitor cells (Lu et al., 2017). It was also found that in Bmi1 knockout neonatal mice, DKK1 expression was significantly upregulated, while β-linked protein and Lgr5 expression were significantly downregulated, suggesting that Bmi1 indirectly exercises its role as an activator of the Wnt signaling pathway by inhibiting the DKK family of Wnt inhibitors (Cho et al., 2013). Bmi1 plays an important role in regulating the proliferation of neonatal mouse cochlear supporting cells and Lgr5-positive progenitor cells through the Wnt signaling pathway, suggesting that Bmi1 may be a new therapeutic target for hair cell regeneration.

### Downregulation of Ephrin-B2 signaling induced trans-differentiation of Kölliker’s organ-supporting cells into hair cells

Ephrins and their Eph receptors are the large Eph family genes that control tissue morphogenesis and are divided into two subclasses, EphB and EphA, based on their affinity for transmembrane EphB ligands or the glycosylphosphatidylinositol-anchored ephrin-A (Gale et al., 1996). EphB and EphA play an important role in the boundary formation between adjacent cell types by interacting with specific EphB or EphA4 receptors (Dahmann et al., 2011). The previous study found that EphA4 receptors interacting with Ephrin-B2 (EphBs at the end of EphA4), is the only receptor to be exclusively expressed in the inner and outer hair cells (Bianchi and Liu, 1999; Miko et al., 2008). While Ephrin-B2 specifically restricted expression in the Kölliker’s organ-supporting cells located medially to the inner hair cell layer from embryonic day (E) 16 and formed a gene-specific expression boundary. Such expression profile can distinguish adjacent supporting cells from hair cells and persists in the post-hearing onset (Defourny et al., 2015). Ephrin-B2 and EphA4 show complementary expression patterns in the developing organ of Corti, participate in sorting at the hair cell/support cell layer and play a key regulatory role in inducing the formation of different cell types in the organ of Corti. It has been hypothesized that cells migrating across non-lineage boundaries transfer their phenotype to fit the identity of their local neighbors, and thus manipulating these boundaries may be an unexpected strategy for generating additional hair cells (Defourny et al., 2011).

Defourny et al. (2015) showed that hair cells could arise directly from Kölliker’s organ-supporting cells located at the medial border with inner hair cell layers by inhibiting Ephrin-B2 signaling. Through using soluble inhibitors, short hairpin RNA (shRNA)-mediated gene silencing, and Ephrin-B2 conditional knockout mice, it was found that at late embryonic stages (the normal hair cell generation phase), downregulation of Ephrin-B2 signaling resulted in translocation of supporting cells to the hair cells layers and subsequent switch in cell identity from supporting cell to hair cell fate. This study demonstrates that manipulation of Ephrin-B2 gene expression in the Kölliker’s organ-supporting cells located at the medial border of the inner hair cell layer at the early stage can serve as a novel hair cell regeneration strategy to directly convert support cells to trans-differentiation hair cells.

Nevertheless, the exact mechanism of this cell fate change precisely occurs remains unclear. One possible explanation is that specific expression of the Sox2 transcription factor in supporting cells induces the differentiation process of supporting cells to hair cells (Millimaki et al., 2010). Furthermore, throughout cochlear development, ephrin-B2 and Notch have similar expression patterns in supporting cell types (Lanford et al., 1999), similar to the vascular regeneration model (Swift and Weinstein, 2009), and Ephrin-B2 may be a downstream target of Notch signaling involved in Notch signaling to couple cell separation and differentiation (Cheng et al., 2004; Tossell et al., 2011). Thus, the downregulation of Ephrin-B2 expression resulting in the absence of Notch signaling pathway components may weaken the fate of supporting cells and make them more susceptible to transition to the hair cell phenotype (Defourny et al., 2015). Further studies are still needed to reveal the underlying mechanisms of this trans-differentiation process.

### Single-cell transcriptome sequencing reveals that Kölliker’s organ-supporting cells have the potential to trans-differentiate into hair cells

Kolla et al. (2020) based on scRNA sequencing technology in E14 and E16 day mouse cochlea found two types of precursor cells expressing Cdkn1b and Sox2 marker genes, which these two groups of cells are located in the medial and lateral regions of the Kölliker’ organ structure, respectively (Kolla et al., 2020). Furthermore, Kubota et al. (2021) used P2 day mice and similarly found that Kölliker’s organ-supporting cell subtypes located in the lateral and medial regions can regenerate into hair cells and supporting cells.

Chen et al. (2021b) also showed that four cell subtypes gradually developed into two different trajectory directions, with one part towards the outer hair cells subtype, and the other part eventually shifting toward the fate of the inner hair cells. The analysis of cell developmental trajectories suggests that these four cell subtypes have the potential to trans-differentiate into inner and outer hair cells, two of which may have the potential to trans-differentiate into outer hair cells, and the other two subtypes have the potential to trans-differentiate into inner hair cells (Chen et al., 2021b).

## Future Research Perspectives and Prospects

Differentiation of Kölliker’s organ-supporting cells to the hair cell phenotype can be induced by overexpression of Math1 and Atoh1 genes, such cells with differentiation potential are fewer and the cell transformation rate is lower (Zheng and Gao, 2000; Shou et al., 2003; Kelly et al., 2012). Therefore, future research should be devoted to improving the efficiency of Kölliker’s organ-supporting cell trans-differentiation into hair cells, and establishing an efficient and safe induction system. Meanwhile, how to induce the transformed neonatal hair cells to form functional mature cilia bundles and establish normal and durable synaptic connections with spiral ganglia (Chai et al., 2017; Li et al., 2019; Xia et al., 2020), to truly achieve functional recovery of neonatal hair cells, needs further study, and immortalized cell lines of hair cell progenitors need to be established.

In the future, there is still a need to optimize the conditions of mouse inner ear stem cell isolated culture and proliferation, to establish a safe and efficient technology system for differentiation of inner ear stem cells into mature hair cells (Wei et al., 2021; Guo et al., 2022; Hu et al., 2022; Tao et al., 2022), to screen mouse stem cell lines that can be used for stem cell therapy (Liu et al., 2018; Tang et al., 2019); discovering new molecular markers specific for inner ear hair cells and their precursor cells (Qi et al., 2019), thus providing conditions for the establishment of rapid and non-destructive cell identification and sorting techniques, and thus laying a solid theoretical foundation and providing technical support for stem cell therapy for sensorineural deafness technical support.

## Author Contributions

JC and DG drafted the manuscript. JY and LS revised the manuscript. All authors contributed to the article and approved the submitted version.

## Funding

This study was funded by the National Natural Science Foundation of China (81873689, 82271179, and 82230035).

## Conflict of Interest

The authors declare that the research was conducted in the absence of any commercial or financial relationships that could be construed as a potential conflict of interest.

## Publisher’s Note

All claims expressed in this article are solely those of the authors and do not necessarily represent those of their affiliated organizations, or those of the publisher, the editors and the reviewers. Any product that may be evaluated in this article, or claim that may be made by its manufacturer, is not guaranteed or endorsed by the publisher.
